# A role for the NLRC4 inflammasome in premature rupture of membrane

**DOI:** 10.1371/journal.pone.0237847

**Published:** 2020-08-24

**Authors:** Jinming Zhu, Chunling Ma, Lina Zhu, Juan Li, Fengyun Peng, Lei Huang, Xiaomei Luan

**Affiliations:** 1 Department of Obstetrics, Xuzhou Maternity and Child Health Care Hospital Affiliated to Xuzhou Medical University, Xuzhou, Jiangsu, China; 2 Graduate School, Xuzhou Medical University, Xuzhou, Jiangsu, China; 3 Department of Obstetrics and Gynecology, Affiliated Huaihai Hospital of Xuzhou Medical University, Xuzhou, Jiangsu, China; University of the Pacific, UNITED STATES

## Abstract

PROM is one of the common complications of perinatal period, which seriously threatens the mother and newborn. The purpose of this study was to identify the role of NLRC4 inflammasomes in this process and their underlying mechanisms. We performed high-throughput RNA sequencing of fetal membrane tissue from 3 normal pregnant women and 3 term-premature rupture of fetal membrane (TPROM) patients who met the inclusion criteria, and found that NLRC4 was significantly up-regulated in TPROM patients. An observational study of TPROM patients (PROM group, n = 30) and normal pregnant women (control group, n = 30) was performed at the Xuzhou Maternal and Child Health Hospital affiliated to Xuzhou Medical University from May 2018 to May 2019. The expression of genes involved in inflammasome complex including NLRC1, NLRC3, AIM2, NLRC4, ASC, caspase-1, IL-6, IL-18 and IL-1βwas determined via real-time PCR, immunohistochemistry and immunofluorescence. Measurement of NLRC4 level in serum was conducted by ELISA assay. The results showed that the NLRC4, ASC, caspase-1, IL-1β and IL-18 levels in fetal membrane, placental tissues and maternal serum were markedly higher in the PROM group than that in the control group. In conclusion, NLRC4 is a markedly up-regulated gene in TPROM fetal membrane tissue, suggesting that NLRC4 is involved in the occurrence and development of TPROM; NLRC4 levels in maternal blood serum are closely related to TPROM and have the potential to assist doctors in predicting and diagnosing PROM.

## Introduction

Premature rupture of membranes (PROM) refers to the rupture of the membranes before delivery, which is characterized by painless fluid leaking from the vagina. It is one of the common complications of perinatal period, which seriously threatens the mother and newborn [1–3]. PROM that occurs prior to 37 weeks of pregnancy is pre-premature rupture of fetal membranes (PPROM) and PROM that occurs after 37 weeks of pregnancy is term-premature rupture of fetal membranes (TPROM). The pathogenesis of TPROM is still unclear, but it usually results from vaginal flora, fallopian tube inflammation, insufficient trophoblastic infiltration, and the abnormal pressure of the amniotic cavity are possible causes of TPROM [4]. At present, clinical prediction of PROM is still difficult to achieve and the effect of traditional single conservative treatment strategies is not satisfactory.

A growing body of evidence supports the concept that physiological and pathological inflammation during pregnancy is linked to the innate immunity. Innate immune receptors, such as C-type lectin receptors (CLR), nucleotide oligomerization domain (NOD)-like receptors (NLR), and absent in melanoma 2 (AIM2)-like receptors (ALR), serve as the first line of defense against infectious microorganisms, constantly monitoring the extracellular milieu and subcellular compartments [5–7].

NLRs are activated by pathogen-associated molecular patterns or by danger-associated signals [8]. The NLR family CARD domain-containing protein 4 (NLRC4) inflammasome, is a multiprotein complex and a member of NLR family, whose activation is closely associated with the secretion of inflammatory factors [9]. It is mainly expressed in macrophages, neutrophils, dendritic cells and glial cells. The inflammasome complex contains a pattern recognition receptor (NLRC4), an adaptor protein (apoptosis-associated speck-like protein containing a CARD, ASC), and pro-caspase-1. Several lines of evidence show that NLRC4 inflammasome promote an inflammatory response through the release of the mature forms of IL-1b and IL-18. In addition, NLRC4 can interact directly with caspase-1 to induce cell death and rapidly initiate inflammation and vascular fluid loss [10–13]. NLRC4 is activated as part of the innate immune response to a range of intracellular bacteria, including S. typhimurium and Legionella pneumophila. Yan Qu et al [14]. proposed that phosphorylation of NLRC4 (S533) can cause rapid macrophage pyroptosis without infection. Furthermore, Bryant AH et al [15]. confirmed that the expression of NLRC4 increases in the placenta and membranes of term pregnancy, but whether it is involved in the process of TPROM is unknown.

The purpose of this study was to identify genes that are differentially expressed in patients with TPROM by a genome-wide approach, and to further explore the role of NLRC4 inflammasomes in this process and their underlying mechanisms.

## Material and methods

### Patients and definitions

This study included 60 pregnant women who delivered at the Xuzhou Maternal and Child Health Hospital affiliated to Xuzhou Medical University from May 2018 to May 2019. Group1 (PROM group) consisted of 30 women with TPROM. Group2 (control group) consisted of 30 women with full-term gestational age≥37 weeks and <42 weeks. All these women had normal outcomes (delivery of a healthy term infant).

All selected women met the following criteria: (a) singleton pregnancy; (b) full-term pregnant women planning to give birth in our hospital. Women with the following conditions were excluded: (a) Women who did not meet the inclusion criteria; (b) patients with severe pregnancy complications; (c) women with neonates having congenital or chromosomal abnormalities. S1 Table includes the demographic and clinical characteristics of the study groups. Membrane rupture was diagnosed with the combination of vaginal pooling, ferning, and nitrazine test.

All women provided informed consent before collection of placental and membrane tissue, and the study was approved by the Clinical Research Ethics Committee of affiliated Hospital of Xuzhou Medical University. We declared that all methods in this study were carried out in accordance with relevant guidelines and regulations.

### RNA-sequencing and bioinformatics

The fetal membrane tissues from 3 normal pregnant women and 3 TPROM patients who met the criteria for inclusion and not combined with histological chorionitis (HCA) were stored in RNA later solution for future study. RNA high throughput sequencing was performed by Cloud-Seq Biotech (Shanghai, China). Briefly, total RNA was used for removing the rRNAs with NEBNext rRNA Depletion Kit (New England Biolabs, Inc., Massachusetts, USA) following the manufacturer's instructions. RNA libraries were constructed by using NEBNext Ultra II Directional RNA Library Prep Kit (New England Biolabs, Inc., Massachusetts, USA) according to the manufacturer’s instructions. Libraries were controlled for quality and quantified using the BioAnalyzer 2100 system (Agilent Technologies, Inc., USA). Library sequencing was performed on an illumina Hiseq instrument with 150 bp paired end reads [16].

### Clinical specimens

Immediately after delivery, the placenta was thoroughly rinsed with sterile ice-cold phosphate buffered saline, cut into appropriately sized pieces (1.0 cm× 1.0 cm× 0.5 cm), and placed in RNAlater solution or 4% paraformaldehyde. The membranes near the breach were peeled from the placentas, and stored at -80°C for further experiments.

### RNA extraction and real-time PCR (RT-PCR)

RNA extraction and Real-time PCR (RT-PCR) were performed as described previously [17]. TRIzol reagent was used to extract RNA from cells and tissues, and the cDNA was generated with random primers (Promega) using the Reverse Transcription System (Promega) was carried out on ABI-7500 using SYBR Green RT-PCR Master Mix (Vazyme Biotech). GAPDH was used for normalization of qRT-PCR data. Primer sequences are available in S2 Table.

### Immunohistochemistry (IHC)

For IHC, after antigen retrieval using EDTA, the specimens were blocked with goat serum for 20 minutes before applying the primary antibody. Specimens were incubated with anti-NLRC4 (Invitrogen, cat #PA5-88997; 1:100) for 12 hours in 4°C. Next, the sections were washed twice and subsequently incubated with HRP-polymer-conjugated secondary antibody (Zhong Shan, China) at room temperature. Finally, the specimens were then stained with 3,3-diaminobenzidine solution and haematoxylin. The slides were photographed with an inverted microscope (Olympus, Japan).

### Immunofluorescence

Immunofluorescence assay was performed as described in research by Hou et al [18]. Sections were permeabilized with Triton X-100 (0.1%) and blocked with solution containing 5% bovine serum before applying primary antibody. Specimens were incubated respectively with anti-NLRC4 (Invitrogen, cat #PA5-88997; 1:100) and anti-caspase-1 (Invitrogen, cat #MA5-32909; 1:100) for 12 hours in 4°C. Secondary antibodies (Life Technologies, catalog #A21207; 1:200) and Alexa Fluor 488 goat anti-mouse IgG (LifeTechnologies, catalog #A11001) were incubated subsequently under light-protected conditions for 1 h at room temperature. Nucleus were staining with DAPI (KeyGen Biotech, catalog #KGA215–10) in the end. After final washing, the cover slips were mounted on slides using 50% glycerin. Then the sections were observed under a fluorescence microscope (Olympus) or confocal laser scanning microscope (Olympus).

### Enzyme-linked immunosorbent (ELISA) assay

Culture medium samples: The culture medium of RAW264.7 cells was collected, and the levels of NLRC4 (Cusabio, cat #CSB-EL015862HU) in the culture medium were detected by enzyme-linked immunosorbent assay. Blood samples: Before labor, 10 ml of peripheral blood was collected in ethylenediaminetetramine Acetic acid (EDTA) vial. Blood samples were centrifuged (600g/min) for 10 minutes, serum was collected and stored at -80 environment for ELISA assay. The levels of NLRC4 (Cusabio, cat #CSB-EL015862HU), IL-1β ((Protein tech, catlog #KE00021), IL-6 (Protein tech, cat #KE00139), IL-18 (Protein tech, catlog # KE00025) and TNF-α ((Protein tech, catlog #KE00154) in the culture medium were detected by enzyme-linked immunosorbent assay

### Statistical analysis

Numerical data are expressed as the mean ± SEM. Two independent sample data sets were tested using 2-tailed Student’s t test. Multiple group comparisons were evaluated by 1-way ANOVA followed by least significant difference t test for post hoc analysis. χ^2^ or Fisher’s exact tests were used to compare categorical variables. Analyses were performed using SPSS software (SPSS, Inc, Chicago, IL). *P*<0.05 was considered as significant difference.

## Results

### Levels of NLRC4 are increased in TPROM

**Fig 1:**
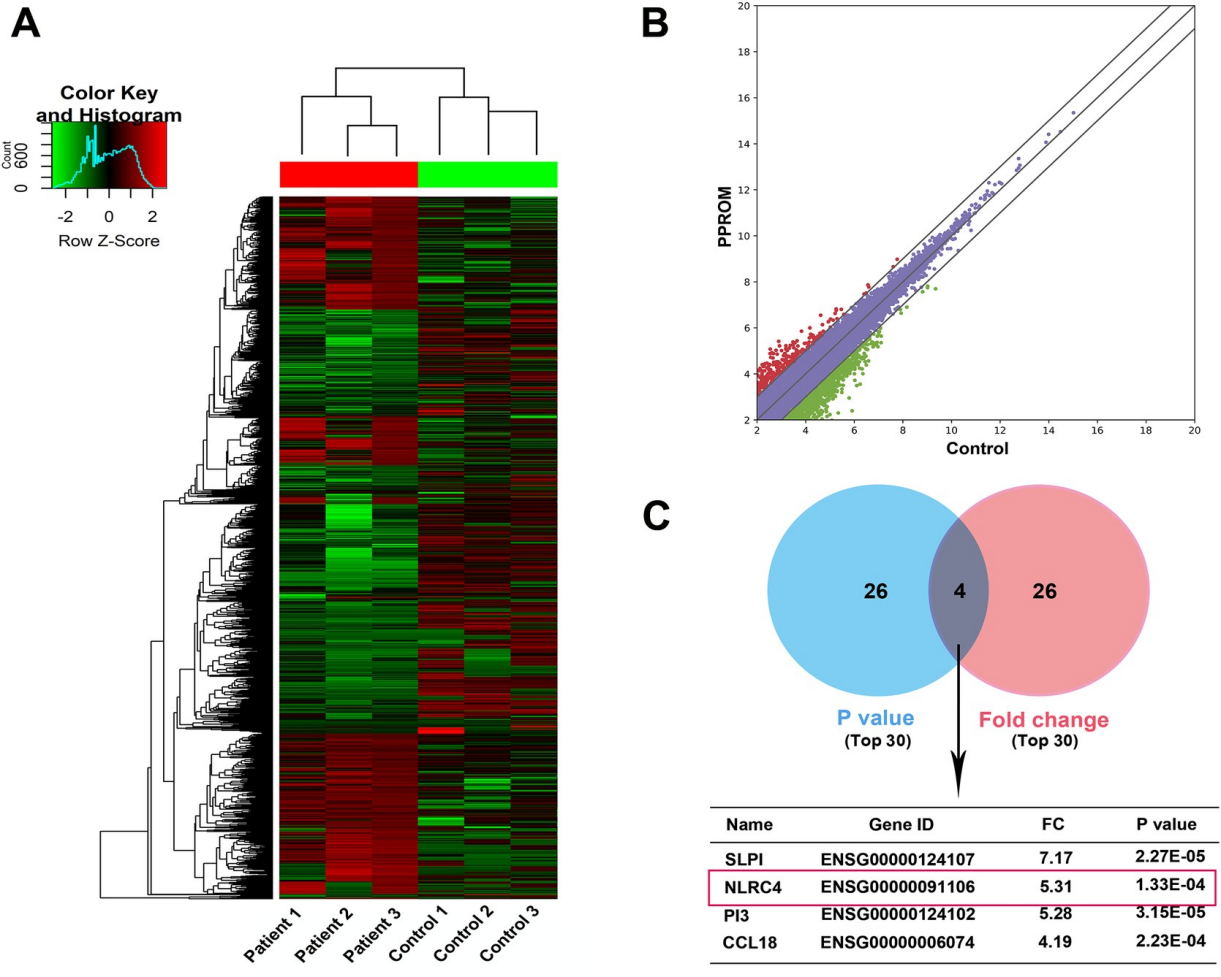
Levels of NLRC4 are increased in PROM. (A) Hierarchical clustering showing differentially expressed mRNAs in membrane tissues of TPROM patients and the normal pregnant (FC< 0.5 or > 2, and *P* < 0.05); (B) Scatter plots of pairwise comparisons between the Control group and the PROM group; (C) Venn diagram about the top 30 gene sets ranked by *P* value or FC.

Hierarchical clustering shows differentially expressed mRNAs in membrane tissues of TPROM patients and the membrane tissues of non-TPROM pregnant (fold change (FC) < 0.5 or > 2, and P < 0.05) (Fig 1A). Scatter plots of pairwise comparisons between the Control group and the PROM group and including 12300 genes are shown in Fig 1B. As shown in the Venn diagram, the overlapping upregulated mRNAs from the top 30 gene sets ranked by P value or FC (Fig 1C). We note that the expression of NLRC4, which is closely related to the inflammatory response, is significantly up-regulated in the fetal membrane tissue of patients with TPROM, so we hypothesized that the NLRC4 are involved in the occurrence and development of PROM.

**Fig 2:**
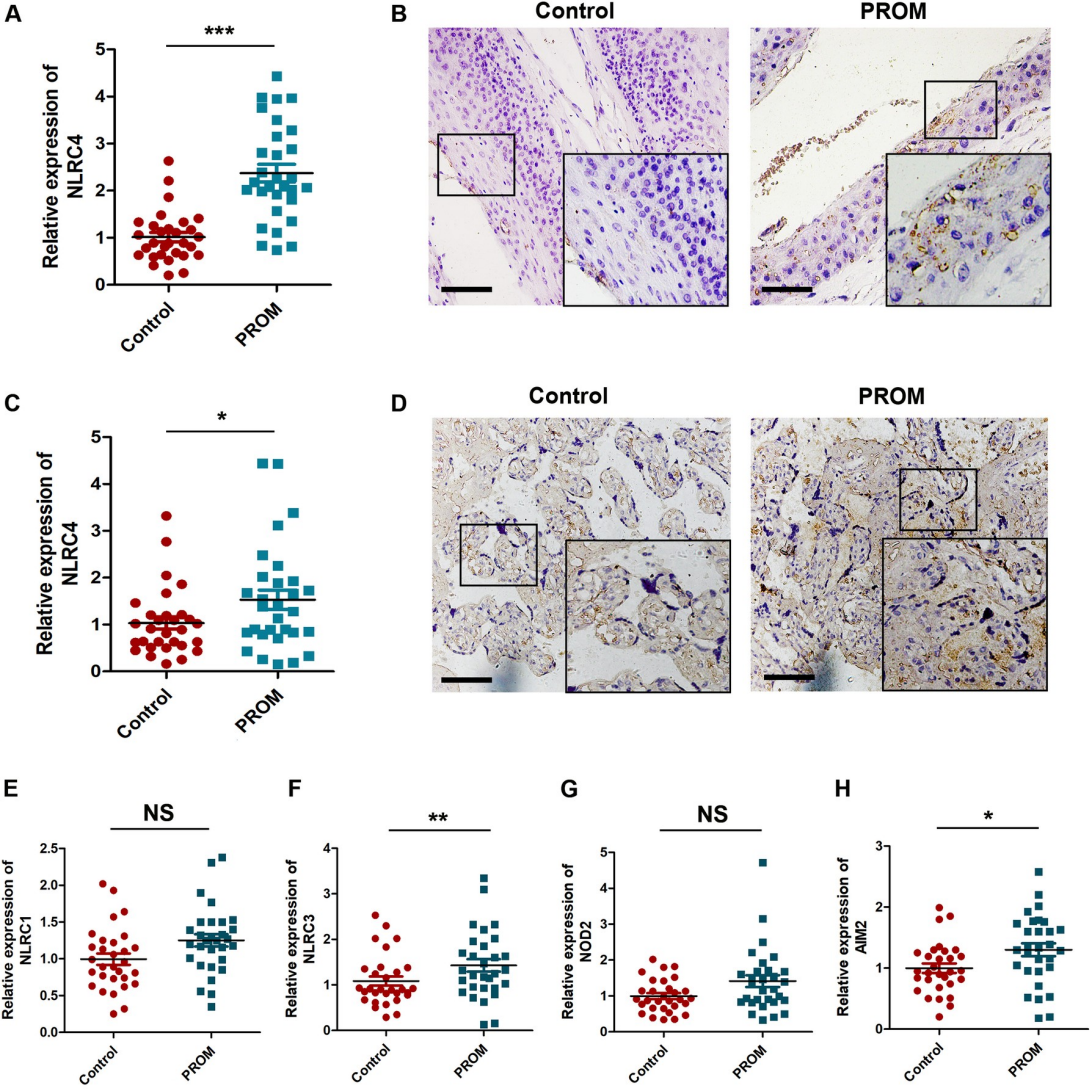
Increased activation of NLRC4 inflammasomes in placenta and membranes of patients with TPROM. (A) RT-PCR analysis of NLRC4 at mRNA level in fetal membrane; (B) NLRC4 immunostaining in fetal membrane was shown, bar = 100 μm; (C) Relative mRNA level of NLRC4 in placenta; (D) Representative IHC staining images of placenta, bar = 100 μm; (E-H) mRNA abundance of inflammasome components and NOD2 in fetal membranes, *P<0.05, **P<0.01, ***P<0.001, NS, *P*>0.05 vs the control group, n = 30.

Next, we designed PCR primers for inflammasomes to specifically detect the RNA expression of NLRC1, NLRC3, NLRC4, NOD2, and AIM2 in the placenta and fetal membranes of 30 cases of TPROM patients and 30 cases of non-TPROM pregnant women. The results indicated that the RNA expression of NLRC4 was significantly increased in both placental (1.53±0.21 versus 1.03±0.13 [control], *P*<0.001) and fetal membrane tissues of patients with TPROM (Fig 2A and 2C). In addition, the RNA expression of NLRC3 (1.25±0.09 versus 0.99±0.15 [control], *P*<0.01) (Fig 2F) and AIM2 (1.30±0.11 versus 1.00±0.08 [control], *P*<0.05) (Fig 2H) inflammasomes in the fetal membranes of patients with TPROM also increased slightly, but there was no difference in the expression of NLRC1(1.19±0.09 versus 1.00±0.13 [control], *P*>0.05) (Fig 2E) and NOD2 (1.39±0.25 versus 0.99±0.18 [control], *P*>0.05) (Fig 2G). Immunohistochemical analysis further confirmed that compared with normal pregnant women, NLRC4 expression was increased in the fetal (Fig 2B) membranes and placenta (Fig 2D) of patients with TPROM.

### NLRC4 inflammasomes activation participates in TPROM process

**Fig 3:**
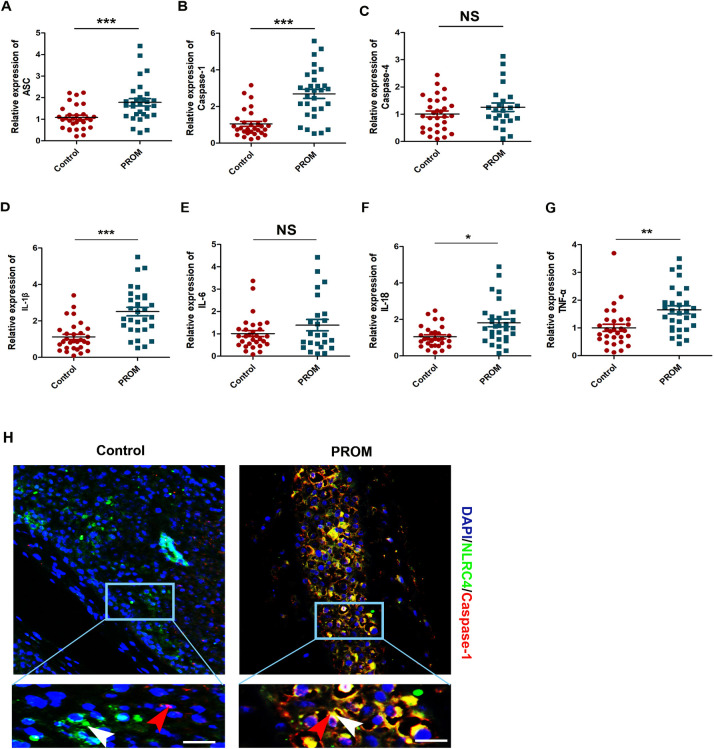
NLRC4 inflammasomes activation participates in TPROM process. (A-G) NLRC4 inflammasomes activation participates in PROM process. ASC, caspase-1, caspase-4, IL-1β, IL-6, IL-18 and TNF-α gene expression in fetal membrane, *P<0.05, **P<0.01, ***P<0.001, NS, *P*>0.05 vs the control group, n = 30; (H) Immunofluorescence analysis of the expression of NLRC4 and caspase-1 in fetal membrane, bar = 20 μm.

NLRC4 inflammasomes activation causes their oligomerization to form multi-protein inflammasome complexes serving as platforms for the recruitment, cleavage, and activation of caspases-1. As shown in Fig 3A, the expression of ASC as inflammasome adaptor protein was significantly increased (1.78±0.29 versus 1.01±0.16 [control], *P*<0.001). Furthermore, compared to the control group, the mRNA abundance of caspase-1 was significantly higher in the fetal membranes of TPROM patients (2.69±0.31 versus 1.05±0.27 [control], *P*<0.001) (Fig 3B). There was no significant difference in the expression of caspase-4 between the PROM group and the control group (1.25±0.19 versus 1.00±0.15 [control], *P*>0.05) (Fig 3C). Immunofluorescence assay showed positive correlation between NRLC4 and caspase-1 expression on fetal membranes. Obviously, fetal membrane tissues with high expression of NLRC4 may recruit more caspase-1 to promote the inflammatory response in patients with TPROM (Fig 3H).

Given that the pathogenesis of PROM is basically related to the inflammatory response and the inflammasome-mediated caspase-1 activation is involved in downstream secretion of the pro-inflammatory cytokines, such as IL-1β and IL-18, we investigated the levels of secreted proinflammatory cytokines in the different groups (Fig 3D–3G). The result indicated that the levels of IL-1β (2.05±0.26 versus 1.03±0.20 [control], *P*<0.01), IL-18 (1.81±0.21 versus 1.04±0.18 [control], *P*<0.01) and TNF-α (1.65±0.15 versus 1.01±0.13 [control], *P*<0.01) in the fetal membranes of patients with TPROM were significantly increased. While there was no significant difference in the expression of IL-6 (1.39±0.25 versus 1.03±0.19 [control], *P*>0.05) in the fetal membranes between the two groups of pregnant women.

In S3 Table, we used the spearman correlation coefficient values to further analyze the correlation of genes involved in inflammasome pathway with PROM. The result indicated that there was a very strong positive correlation between the NLRC4 (r = 0.82, *P* = 0.01) and caspase-1 (r = 0.70, *P* = 0.03) gene expression with PROM.

### Evaluation of NLRC4 in maternal blood serum for prediction of PROM

**Fig 4:**
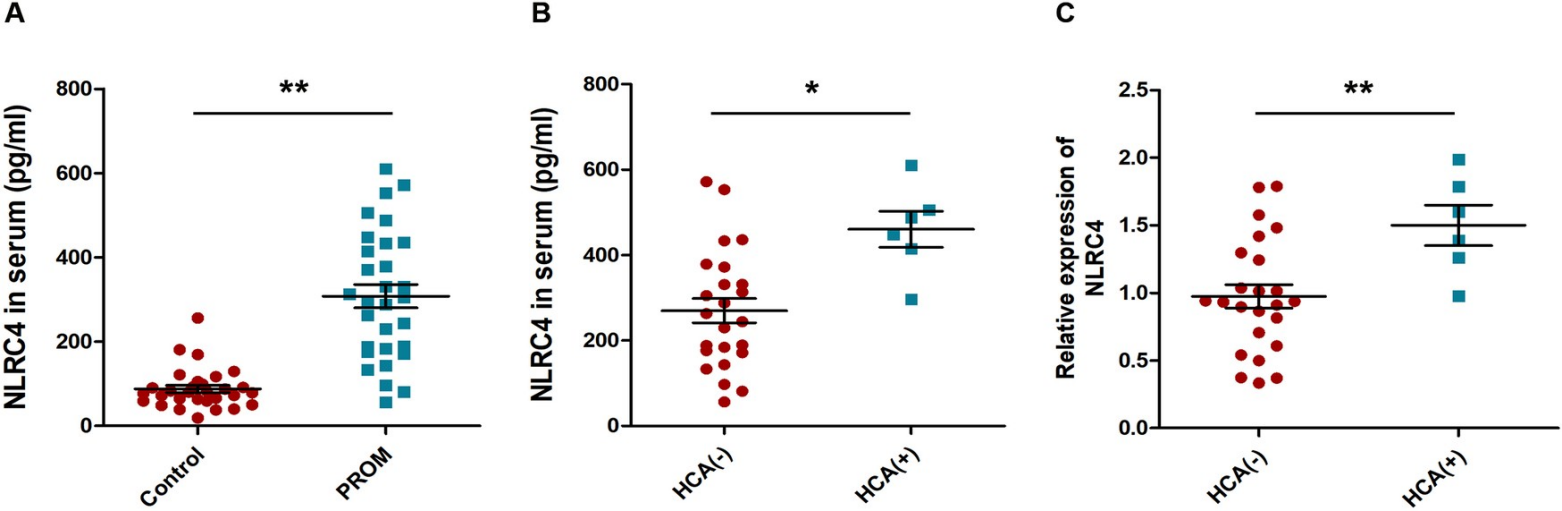
Evaluation of NLRC4 in Maternal Blood serum for Prediction of PROM. (A) ELISA assay of the NLRC4 level in maternal blood serum, **P<0.01 vs the indicated group, n = 30; (B, C) Detection of NLRC4 level in PROM patients and PROM combined with HCA patients by ELSIA assay and RT-PCR, *P<0.05, **P<0.01 vs the indicated group, n = 30.

To further understand whether the level of NLRC4 in maternal blood is related to the onset of PROM, the NLRC4 levels were measured by ELISA in the serum samples collected from TPROM patients and control study subjects. The results indicated that NLRC4 level in maternal blood was significantly different in the patients with TPROM and non-TPROM pregnant (307.9±27.8 versus 87.97±14.3 [control], *P*<0.01) (Fig 4A).

It is worth noting that 6 women in the PROM group were diagnosed with TPROM combined with Histological chorioamnionitis (HCA). Considering that HCA is the main cause and common complication of PROM, we further analyzed the relationship between HCA and NLRC4 content in serum of patients with TPROM. As shown in Fig 4B and 4C, the NLRC4 level in both serum (460.2±62.3 versus 289±55.1 [control], *P*<0.05) and fetal membranes (1.50±0.16 versus 0.99±0.08 [control], *P*<0.01) of patients with TPROM combined with HCA was slightly higher than that of patients with TPROM with non-HCA. Overall, we conclude that high levels of NLRC4 may be one of the hallmarks of PROM.

## Discussion

The main findings of this study are that NLRC4 plays an important role in the occurrence and development of TPROM, and that the level of NLRC4 in maternal serum is of great value in predicting the onset of TPROM.

PROM not only causes chorioamnionitis, septicemia, placental abruption, and endometritis, but premature birth caused by PROM is also harmful to the newborn's health, such as causing neonatal ventricular bleeding, softening of white matter around the ventricle, and cerebral palsy [19, 20]. Therefore, fully understanding the pathological mechanism of TPROM is of great significance for the early diagnosis and early treatment of TPROM and improving the perinatal outcome of mothers and infants. However, the lack of typical clinical symptoms and sensitive diagnostic methods before the onset of PROM greatly increases the difficulty for obstetricians to cope with and manage premature rupture of membranes.

Amniocentesis and amniotic fluid culture are recommended by the American Academy of Obstetrics and Gynecology for diagnosing amnion infection and indirectly for predicting PROM [21]. However, limitations such as their invasiveness and low positive detection rate have determined that their use in predicting TPROM is not widely accepted. In this study, we used RNA high throughput sequencing as a screening tool to select genes for more detailed and quantitative analyses, and found that NLRC4 is a generally up-regulated gene in the membranes of patients with TPROM. Statistical analysis of mRNA levels identified by PCR and protein levels identified by IHC were altered to the same direction. Gomez-Lopez N et al [22] proposed that NLRC4 expression is increased in infected fetal membrane tissues, but whether NLRC4 is involved in childbirth, especially in PROM, has not been reported. We also found that NLRC4 expression is up-regulated not only in placental tissues, but also in placenta and maternal serum of patients with TPROM. Consequently, we propose that the level of NLRC4 in maternal blood serum can be used as an indicator to assist in the prediction and diagnosis of TPROM. In summary, we believed that NLRC4 has the potential to be a valuable auxiliary examination item for obstetricians when managing TPROM.

The mechanisms responsible for TPROM have not been elucidated, but are thought to involve inflammation. In addition to NLRC4, we also observed upregulation of caspase-1, ASC, IL-1β, and IL-18 in the membranes of patients with TPROM. In mechanism, the classic NLRC4 inflammasome pathway, that is, after binding to the ligands, the NLRC4 inflammasome is activated, resulting in its oligomerization to form multi-protein inflammasome complexes, which serves as a platform for recruitment, cleavage, and activation of caspase-1. Caspase-1 would then cleave pro-IL-1b, pro-IL-18, into their mature secreted forms of the cytokines [23–25]. Here, our results demonstrated that NLRC4 was upregulated in the membranes of patients with TPROM and recruited more caspase-1. Not surprisingly, the expression of IL-1β, IL-18 and TNF-α in fetal membrane tissue and maternal serum also increased at the RNA and protein levels. It is well known that IL-1 β and TNF—α are closely related to cell apoptosis, and IL-1β can up regulate the expression of matrix metalloproteinases (MMPs) in fetal membranes, and then degrade the extracellular matrix, resulting in rupture of fetal membranes [26–28]. Therefore, we speculated that NLRC4 promotes the process of rupture of fetal membranes by inducing apoptosis and degrading ECM. Based on the above findings, we believed that NLRC4-mediated inflammation plays an important role in the pathogenesis of TPROM.

NLRs are usually activated by pathogen-associated molecules patterns. TPROM combined with HCA is not uncommon in obstetrics [29, 30]. In this study, 6 TPROM patients were diagnosed with HCA after delivery. We performed a subgroup analysis of 30 TPROM patients included in this study and the results indicated that HCA can further promote the NLRC4 level in placenta and serum of patients with TPROM. However, none of the 30 women in the control group suffered from HCA. Therefore, it is still unknown whether HCA can independently upregulate NLRC4 expression and activate the NLRC4 inflammasome pathway.

## Conclusion

In summary, NLRC4 is a markedly up-regulated gene in TPROM fetal membrane tissue, suggesting that NLRC4 is involved in the occurrence and development of TPROM. In addition, NLRC4 levels in maternal blood serum are closely related to TPROM and have the potential to assist doctors in predicting and diagnosing PROM

## Supporting information

S1 Table Characteristics of cohort: Demographics and procedure indications. (DOCX)

S2 Table Primer sequences used in this study. (DOCX)

S3 Table Expression of genes involved in inflammasome pathway. (DOCX)
