# Supplementary material for: A role for the NLRC4 inflammasome in premature rupture of membrane
Source: PLoS One. 2020 Aug 24;15(8):e0237847. doi: 10.1371/journal.pone.0237847 (PMC7446792; doi:10.1371/journal.pone.0237847)
Supplement: S1 Table — (DOCX) [file pone.0237847.s001.docx]

**S1 Table: Characteristics of cohort: demographics and procedure indications**

|  | **Term controls (n = 30)** | **TPROM cases (n = 30)** | **P value** |
| --- | --- | --- | --- |
| Maternal age (years) | 30.40 ± 4.62 | 30.10 ± 3.57 | 0.69 |
| Nulliparous | 11 (37%) | 8 (28%) | 0.58 |
| Prior abortion | 6 (20%) | 7 (23%) | 0.76 |
| Body mass index (kg/m2) | 28.15 ± 3.63 | 26.36 ± 2.51 | 0.23 |
| Birth weight (g) | 2983 ± 763 | 2793 ± 669 | 0.33 |
| Gestational age at birth (weeks) | 38.97 ± 1.12 | 39.23 ± 0.85 | 0.58 |
| Chorioamnionitis | 0 (0%) | 6 (20%) | 0.02* |

TPROM = Time-premature rupture of fetal membranes；Data presented as mean ± SD or as a proportion of n；*P＜0.05 vs the control group.
