# Supplementary material for: A role for the NLRC4 inflammasome in premature rupture of membrane
Source: PLoS One. 2020 Aug 24;15(8):e0237847. doi: 10.1371/journal.pone.0237847 (PMC7446792; doi:10.1371/journal.pone.0237847)
Supplement: S2 Table — (DOCX) [file pone.0237847.s002.docx]

**S2 Table: Primer sequences used in this study**

| Gene | Forward (5’-3’) | Reverse (5’-3’) |
| --- | --- | --- |
| NLRC1 | GCTTCAGCCCCCAAAGAT | TTGTCCAAGAGAGGGTCCAC |
| NLRC3 | GCTGAACTTGAGCAACAACG | CACCCAACTGTAGGCTCTGC |
| NLRC4 | GCCGGAAGTGAAGCTCTA | CCCCTCCAGTTGCTTCAG |
| AIM2 | TGGAAACCAGAGCAAAACAA | TGGGCTTTGCAGCCTTAATA |
| NOD2 | TAAGAATGCCCGCAAGGTG | GCAGAAAGTGCCGCAGGAT |
| ASC | GACAGTACCAGGCAGTTCG | GTCACCAAGTAGGGCTGTGT |
| Caspase-1 | ACATCTTTCTCCGAGGGTTG | CACCTCTTTCACCATCTCCAG |
| Caspase-4 | AAAGGAGAGAAACAACCGCACAC | TCGGAGGCAGATGGTCAAAC |
| IL-1β | GAGTCTGCACAGTTCCCCAA | TGTCCCGACCATTGCTGTT |
| IL-6 | TAGTCCTTCCTACCCCAATTT | TTGGTCCTTAGCCACTCCTTC |
| IL-18 | CCTGATATCGACCGAACAGC | CCTTCCATCCTTCACAGATAGG |
| TNF-α | GCTGCACTTTGGAGTGATCG | TCAGCTTGAGGGTTTGCTACA |
| GAPDH | TCAAGAAGGTGGTGAAGCAG | AGGTGGAAGAATGGGAGTTG |
