# Supplementary material for: A role for the NLRC4 inflammasome in premature rupture of membrane
Source: PLoS One. 2020 Aug 24;15(8):e0237847. doi: 10.1371/journal.pone.0237847 (PMC7446792; doi:10.1371/journal.pone.0237847)
Supplement: S3 Table — (DOCX) [file pone.0237847.s003.docx]

**S3 Table: Expression of genes involved in inflammasome pathway**

|  | **r** | **P value** |
| --- | --- | --- |
| **NLRC1** | **-0.09** | **0.81** |
| **NLRC3** | **-0.37** | **0.33** |
| **NLRC4** | **-0.82** | **0.01*** |
| **NOD2** | **0.37** | **0.42** |
| **AIM2** | **0.66** | **0.19** |
| **Caspase-1** | **-0.79** | **0.03*** |
| **ASC** | **-0.80** | **0.09*** |
| **IL-1β** | **-0.51** | **0.17** |
| **IL-18** | **-0.44** | **0.29** |
| **TNF-α** | **-0.48** | **0.35** |

*P＜0.05 vs the control group.
